# *Aspergillus* Galactosaminogalactan Mediates Adherence to Host Constituents and Conceals Hyphal β-Glucan from the Immune System

**DOI:** 10.1371/journal.ppat.1003575

**Published:** 2013-08-22

**Authors:** Fabrice N. Gravelat, Anne Beauvais, Hong Liu, Mark J. Lee, Brendan D. Snarr, Dan Chen, Wenjie Xu, Ilia Kravtsov, Christopher M. Q. Hoareau, Ghyslaine Vanier, Mirjam Urb, Paolo Campoli, Qusai Al Abdallah, Melanie Lehoux, Josée C. Chabot, Marie-Claude Ouimet, Stefanie D. Baptista, Jörg H. Fritz, William C. Nierman, Jean Paul Latgé, Aaron P. Mitchell, Scott G. Filler, Thierry Fontaine, Donald C. Sheppard

**Affiliations:** 1 Departments of Microbiology and Immunology, Medicine, McGill University, Montréal, Québec, Canada; 2 Aspergillus Unit, Institut Pasteur, Paris, France; 3 Division of Infectious Diseases, Los Angeles Biomedical Research Institute at Harbor - University of California, Los Angeles Medical Center, Torrance, California, United States of America; 4 J. Craig Ventker Institute, Rockville, Maryland, United States of America; 5 Biological Sciences, Carnegie Mellon University, Pittsburgh, Pennsylvania, United States of America; 6 David Geffen School of Medicine at University of California, Los Angeles, Los Angeles, California, United States of America; Washington University School of Medicine, United States of America

## Abstract

*Aspergillus fumigatus* is the most common cause of invasive mold disease in humans. The mechanisms underlying the adherence of this mold to host cells and macromolecules have remained elusive. Using mutants with different adhesive properties and comparative transcriptomics, we discovered that the gene *uge3*, encoding a fungal epimerase, is required for adherence through mediating the synthesis of galactosaminogalactan. Galactosaminogalactan functions as the dominant adhesin of *A. fumigatus* and mediates adherence to plastic, fibronectin, and epithelial cells. In addition, galactosaminogalactan suppresses host inflammatory responses in vitro and in vivo, in part through masking cell wall β-glucans from recognition by dectin-1. Finally, galactosaminogalactan is essential for full virulence in two murine models of invasive aspergillosis. Collectively these data establish a role for galactosaminogalactan as a pivotal bifunctional virulence factor in the pathogenesis of invasive aspergillosis.

## Author Summary

Invasive aspergillosis is the most common mold infection in humans, predominately affecting immunocompromised patients. The mechanisms by which the mold *Aspergillus fumigatus* adheres to host tissues and causes disease are poorly understood. In this report, we compared mutants of *Aspergillus* with different adhesive properties to identify fungal factors involved in adherence to host cells. This approach identified a cell wall associated polysaccharide, galactosaminogalactan, which is required for adherence to a wide variety of substrates. Galactosaminogalactan was also observed to suppress inflammation by concealing β-glucans, key pattern associated microbial pattern molecules in *Aspergillus* hyphae, from recognition by the innate immune system. Mutants that were deficient in galactosaminogalactan were less virulent in mouse models of invasive aspergillosis. These data identify a bifunctional role for galactosaminogalactan in the pathogenesis of invasive aspergillosis, and suggest that it may serve as a useful target for antifungal therapy.

## Introduction

The incidence of invasive mold infections due to the fungus *Aspergillus fumigatus* has increased dramatically in hematology patients receiving intensive cytotoxic chemotherapy or undergoing hematopoietic stem cell transplantation [1]. Despite the advent of new antifungal therapies, the mortality of invasive aspergillosis (IA) remains 60–80% [2]. There is therefore a pressing need for novel therapeutic strategies to treat or prevent IA. A better understanding of the pathogenesis of IA is one approach that may inform the development of new therapeutic targets.

Adherence of *A. fumigatus* to host constituents is thought to be an early and critical step in the initiation of colonization and infection [3]. Upon inhalation, *A. fumigatus* conidia rapidly adhere to pulmonary epithelial cells and resident macrophages before being internalized and germinating within host cells [4], [5], [6]. Following germination, filamentous hyphae remain in intimate contact with host epithelial, endothelial and immune cells and can induce tissue injury and inflammatory responses. Inhibition of these adherence events may provide a useful therapeutic strategy to reduce morbidity and mortality of *A. fumigatus* mediated disease.

Despite the fact that hyphae play such a critical role in the pathogenesis of invasive aspergillosis, the fungal ligands governing adherence of *A. fumigatus* hyphae to host constituents are largely unknown. A bioinformatic analysis of potential adhesins of *A. fumigatus* has identified several candidate proteins involved in mediating adhesion to host constituents [7], but the adherence of mutant strains that lack these proteins has not been reported. Carbohydrate constituents of the cell wall have been recently implicated in adherence events [8] although their role in mediating adherence to host constituents has not been studied.

We recently identified a fungal regulatory protein, MedA, which governs fungal adhesion to host cells and basement membrane constituents and biofilm formation [9]. In addition, we found that a strain deficient in StuA, a previously described developmental transcription factor [10], was similarly deficient in the formation of adherent biofilms. In contrast deletion of *ugm1*, which encodes a UDP-galactose mutase required for the production of galactofuranose, has been reported to result in a strain of *A. fumigatus* with increased adherence to epithelial cells [11]. Here, we report that carbohydrate analysis of these mutants revealed that the Δ*medA* and Δ*stuA* mutants were defective in galactosaminogalactan (GAG) production whereas the Δ*ugm1* mutant hyperproduced GAG. A comparative transcriptome analysis of the Δ*medA* and Δ*stuA* regulatory mutants identified a gene encoding a putative UDP-glucose-epimerase, designated *uge3*, which was dysregulated in both the Δ*stuA* and Δ*medA* mutants. Disruption of *uge3* resulted in a complete block in GAG synthesis, and markedly decreased adhesion to host cells and biofilm formation. The Uge3 deficient strain was also attenuated in virulence and induced a hyperinflammatory response in a corticosteroid treated mouse model. The absence of GAG in hyphae resulted in an increased exposure of cell wall β-glucan and in higher levels of dectin-1 binding, in association with the release of higher levels of pro-inflammatory cytokine by dendritic cells. Blocking dectin-1 with an anti-dectin-1 antibody, or pre-incubating hyphae with Fc-dectin-1 blocked this increased cytokine production. Suppression of inflammation in mice treated with cyclophosphamide and cortisone acetate resulted in further attenuation of virulence of the Δ*uge3* mutant, although the degree of reduction in fungal burden was similar in neutropenic and corticosteroid treated mice. Collectively these data identify GAG as a multifunctional virulence factor that mediates adherence of *A. fumigatus*, cloaks β-glucan and suppresses host inflammatory responses in vivo.

## Results

### Identification of galactosaminogalactan as a major adhesin of *A. fumigatus*

**Figure 1:**
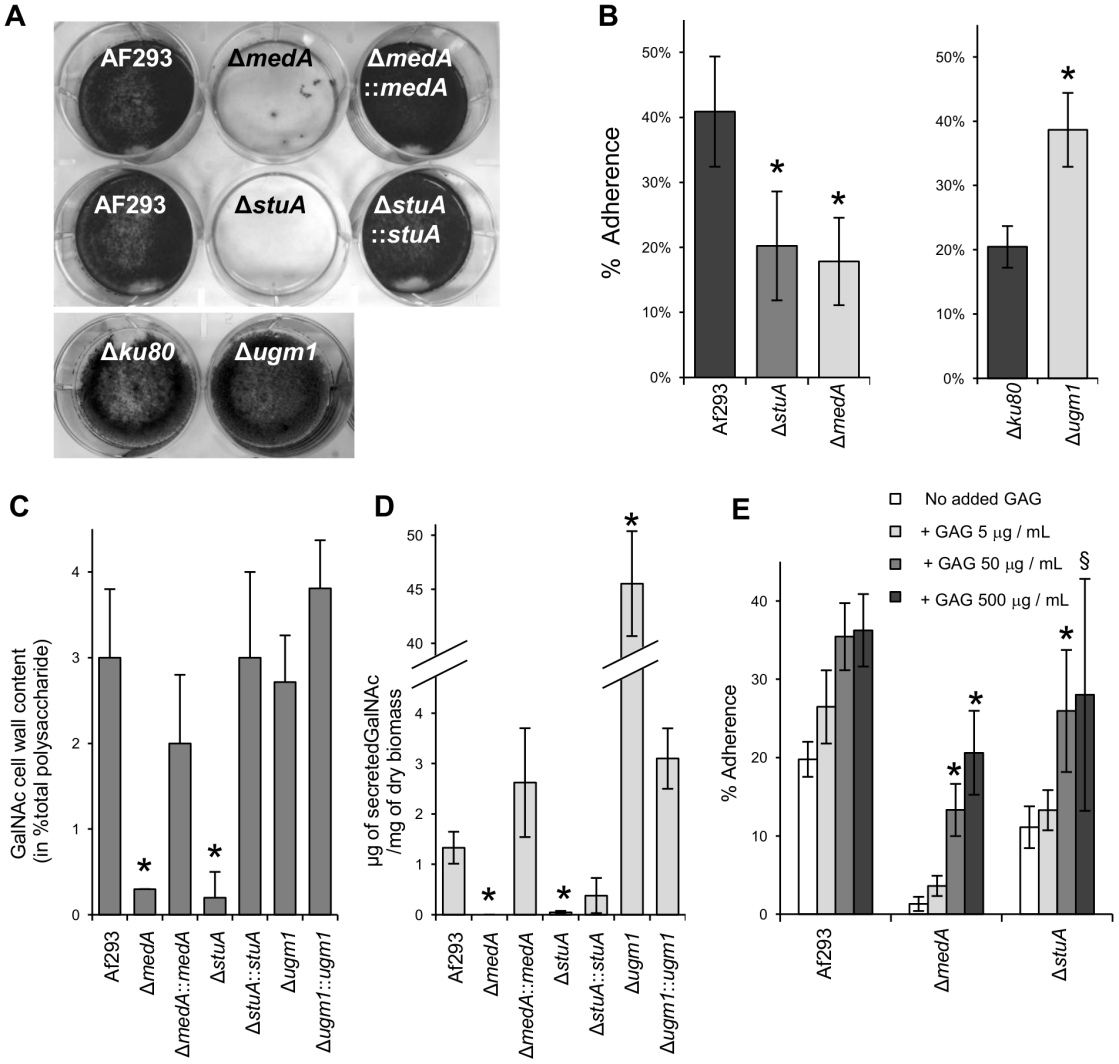
MedA and StuA are required for biofilm formation, adherence to plastic, and galactosaminogalactan (GAG) production. (A) Formation of biofilms by hyphae of the indicated strains after 24 h growth on polystyrene plates. After washing, hyphae were stained with crystal violet for visualization. (B) Adherence of germlings of the indicated strains to polystyrene plates. (C) GalNAc content of the hyphal cell wall of the indicated *A. fumigatus* strains. Results are expressed as a percentage of the total carbohydrate content of the alkali insoluble fraction of the cell wall. (D) Amount of GAG released in the culture supernatant of the indicated strains after 48 h growth, normalized to the culture dry weight. (E) Effects of GAG supplementation on germling adherence of the indicated strains. 6-well polystyrene culture plates were coated with GAG extracted from Af293 culture at the indicated concentration overnight at 4°C before testing the adherence of germlings. All graphs indicate mean ± standard error and represent data obtained from at least three independent experiments performed on separate days. For graphs (B–D) *indicates a significant reduction as compared with the wild-type Af293 strain, with *p* value<0.05 by factor ANOVA. For graph (E): * and ^§^ indicate increased adherence of strains with the addition of GAG with *p* values of <0.05 and  = 0.08, respectively, by factor ANOVA.

Previously, a mutant deficient in UDP-galactofuranose mutase (*ugm1*) was reported to have increased adherence to host cells and abiotic substrates, while an *A. fumigatus* mutant deficient in the regulatory factor MedA had markedly impaired adherence to multiple substrates and was defective in biofilm formation [9]. To identify other mutants with alterations in adhesion, we screened a collection of regulatory mutants including mutants deficient in StuA, BrlA, AcuM and DvrA [10], [12], [13], [14] for their ability to form biofilms on plastic surfaces. Using this approach, we found that the Δ*stuA* mutant previously described by our group [10] was also markedly impaired in the formation of adherent biofilms on plastic (Fig. 1A, 1B). We hypothesized that these differences in the adherence properties of these three strains might stem from alterations in expression of a single adhesion factor. To test this hypothesis, we performed an analysis of the cell wall carbohydrate composition of these mutant strains in comparison to their respective complemented strains and wild-type *A. fumigatus*. The cell walls of the hypoadherent Δ*medA* and Δ*stuA* strains, but not the hyperadherent Δ*ugm1* strain, were found to contain a significant reduction in N-acetyl galactosamine (GalNAc) (Fig. 1C). Since N-acetyl galactosamine is a key component of galactosaminogalactan (GAG), a glycan found within the amorphous cell wall and extracellular matrix of *A. fumigatus* during infection [15], [16], these results suggested that GAG is involved in the differential adhesive properties of these mutants. Consistent with this hypothesis, culture supernatants from the Δ*medA* mutant contained no detectable GAG, while only trace amounts of GAG were found in supernatants from the Δ*stuA* mutants (Fig. 1D). In contrast, culture supernatants from the Δ*ugm1* mutant contained markedly increased GAG as compared to the wild-type and *ugm1* complemented strains.

To test the hypothesis that GAG mediates *A. fumigatus* adherence, we examined the ability of a suspension of extracellular GAG harvested from wild-type hyphae to rescue the adherence defects of the Δ*stuA* and Δ*medA* hyphae. The addition of supplemental GAG resulted in a dose dependent increase in adherence to tissue culture treated plates (Fig. 1E). The addition of exogenous GAG also increased the adherence of wild-type *A. fumigatus*, although to a lesser extent than was seen with the adhesion deficient mutants treated with GAG. Collectively these results suggested that GAG was responsible for the adherence of *A. fumigatus* to plastic and other substrates.

### Comparative transcriptome analysis of Δ*medA* and Δ*stuA* strains identifies a co-regulated gene *uge3*, encoding a candidate GAG biosynthetic enzyme

**Figure 2:**
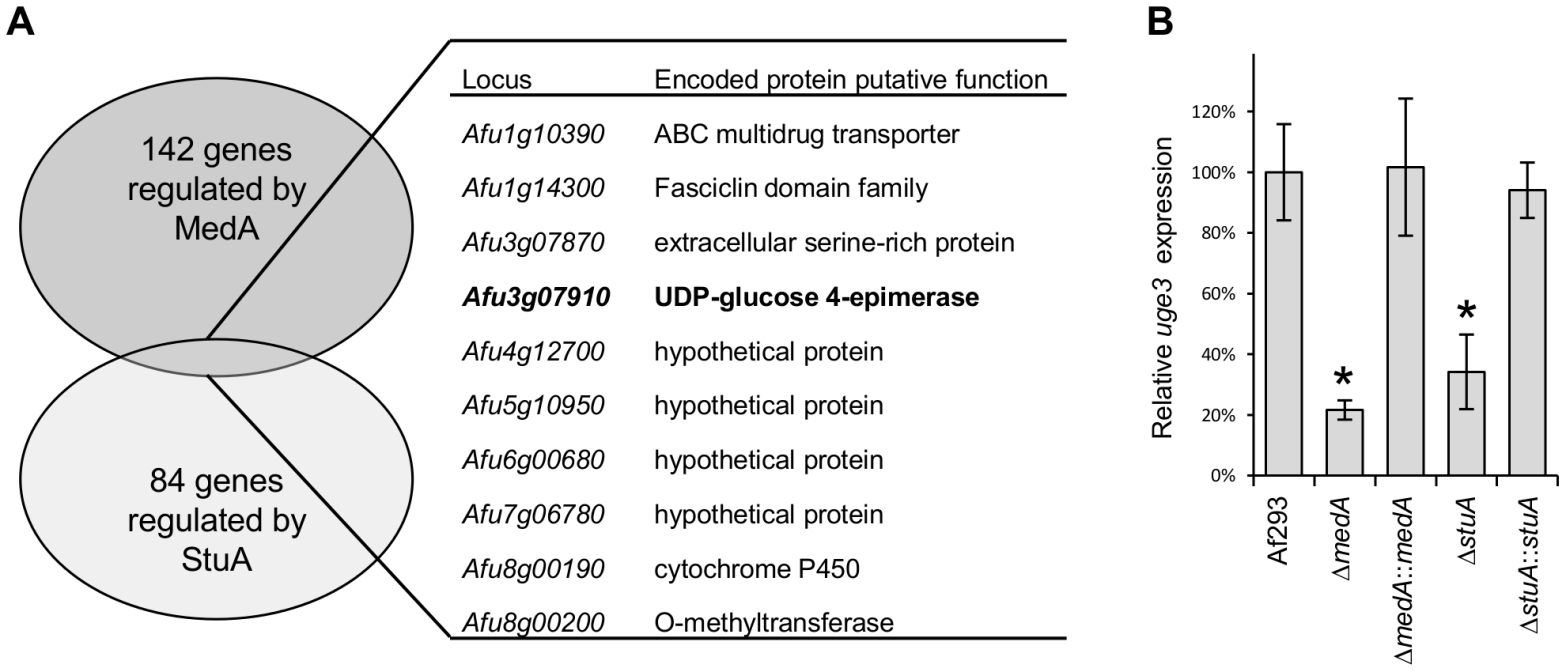
The gene *uge3 (Afu3g07910)* encodes a UDP-glucose-4-epimerase and is regulated by both MedA and StuA. (A) Genes found to be differentially regulated in both Δ*medA* and Δ*stuA* strains by microarray analysis. (B) Expression of *uge3*, measured by real-time RT-PCR in the indicated strains after 18 h growth. Results indicate the mean expression level from at least three independent experiments ± standard error. * indicates a significantly reduced *uge3* mRNA levels as compared with Af293 strain, *p*<0.05 by factor ANOVA.

Since MedA and StuA control the expression of hundreds of genes, to remove any pleiotrophic effect and to test the specific role of GAG production in adherence, we sought to identify the specific genes required for GAG synthesis. Whole genome microarray analysis of the Δ*medA* strain was performed during hyphal growth and development, and compared with the wild-type and *medA* complemented strain. Genes that were significantly dysregulated in the Δ*medA* strain were then compared with the list of previously identified Δ*stuA* dependent genes [10]. Ten genes were identified as being significantly dysregulated in both mutant strains (Fig. 2A). Among these genes was *Afu3g07910*, predicted to encode a UDP-glucose epimerase. Given the role of glucose epimerases in the biosynthesis of galactose and galactosamine, this gene, designated *uge3*, was selected for further study. Real-time RT-PCR confirmed that *uge3* expression was reduced in both the Δ*stuA* and Δ*medA* strains (Fig. 2B).

### Disruption of *uge3* specifically abrogates GAG production

**Figure 3:**
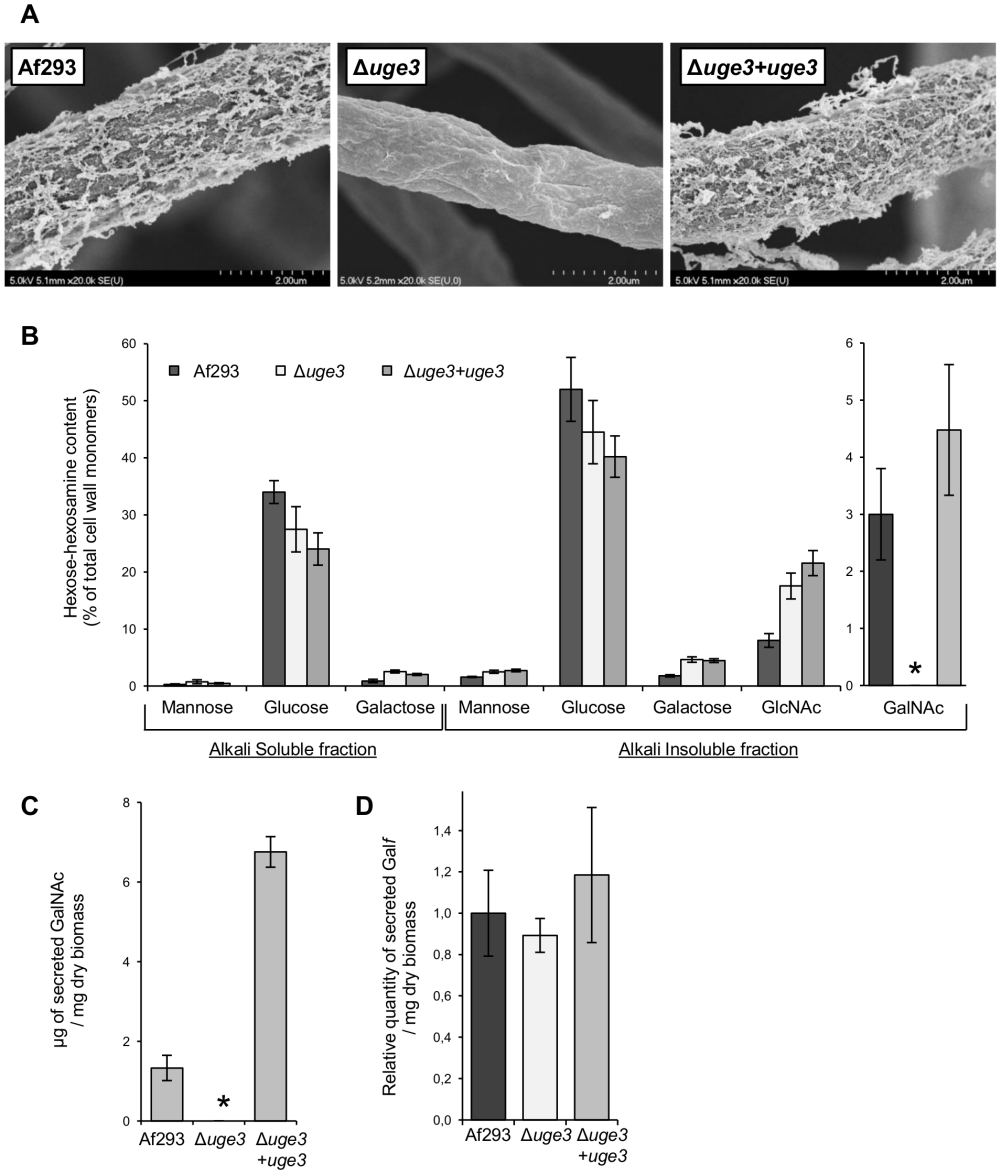
Uge3 is necessary for the production of GAG but dispensable for galactofuranose synthesis. (A) Scanning electron micrographs of hyphae of the indicated strains after 24 h of growth. Magnification was 20,000×. (B) Hexose and hexosamine content of the hyphal cell wall of the indicated *A. fumigatus* strains. Results are expressed as a percentage of the total carbohydrate content of the cell wall. Quantity of GalNAc (C) and of galacto-furanose (Gal*f*) (D) released in the culture supernatant of the indicated strains after 48 h growth, normalized to the culture dry weight. Graphs B, C and D indicate the mean ± standard error of at least three independent experiments. * indicates a significant reduction as compared with the wild-type Af293 strain, *p*<0.05 by factor ANOVA.

To test the role of Uge3 in the synthesis of GAG, a Δ*uge3* mutant strain was constructed. Deletion of *uge3* had no observable effects on growth or morphology including conidiation, conidia size, germination, and radial hyphal growth on solid media in a wide variety of conditions including: minimal media, nutrient rich media (YPD), pH range 4.5 to 8.5, varying iron concentrations (from 0 to 30 µM), and microaerophilic or normoxic conditions (Fig. S1). Scanning electron microscopy of Δ*uge3* mutant hyphae revealed a complete loss of surface decoration and of intercellular matrix (Fig. 3A). Cell wall analysis of the Δ*uge3* mutant strain demonstrated an undetectable level of N-acetyl galactosamine (Fig. 3B), and no GAG was detected in culture filtrates from this strain (Fig. 3C). The production of soluble galactofuranose was unaffected (Fig. 3D). When compared with wild-type *A. fumigatus*, a slight increase in cell wall GlcNAc content was observed in the Δ*uge3* mutant (Fig. 3B), as well as a minimally increased resistance to the anti-chitin agent nikkomycin but not to the chitin binding agent calcofluor white (Table 1). However, complementation of the Δ*uge3* mutant with an intact allele of *uge3* had no effect on these observations, despite completely restoring N-acetyl galactosamine and GAG synthesis. Collectively these results suggest that Uge3 is necessary for the production of GAG.

**Table 1:** Sensitivity of fungal strains to cell wall perturbing agents.

Fungal strain	Minimum Inhibitory/Effective Concentration (µg/mL)		
	Caspofungin	Nikkomycin	Calcofluor White
Af293	0,42	1,12	10,42
Δ*uge3*	0,50	1,92	10,42
Δ*uge3*+*uge3*	0,42	1,92	10,42

### The *uge3* deficient mutant is markedly deficient in adherence to and damage of pulmonary epithelial cells

**Figure 4:**
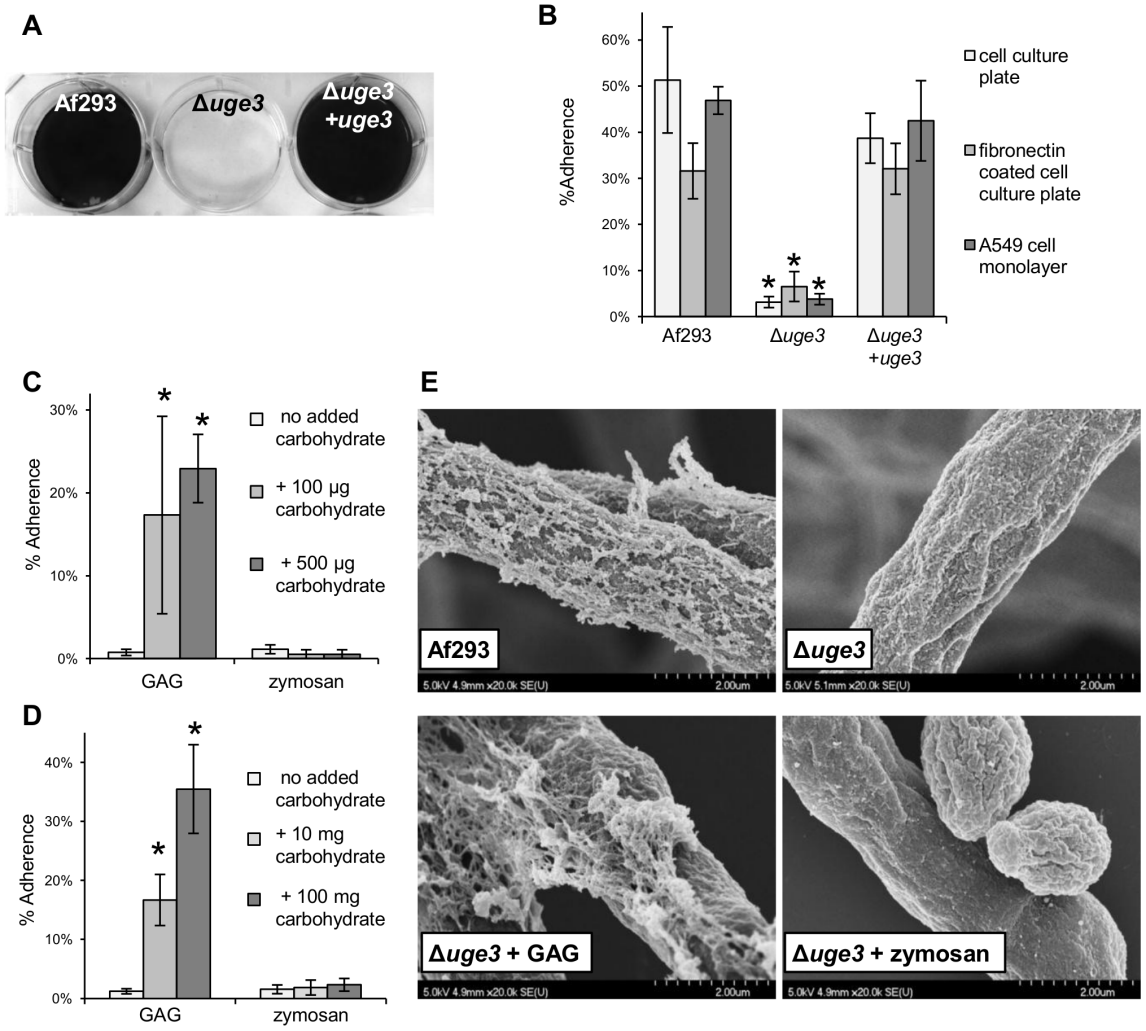
Uge3 is necessary for normal adherence of *A. fumigatus* hyphae. (A) Formation of biofilms by hyphae of the indicated strains after 24 h growth on polystyrene plates. After washing, hyphae were stained with crystal violet for visualization. (B) Adherence of germlings of the indicated strains to cell culture treated polystyrene, fibronectin and A549 epithelial cells. (C) Adherence of the Δ*uge3* strain to cell culture treated polystyrene supplemented with a suspension of GAG or zymosan at the indicated concentrations. (D) Adherence to cell culture treated polystyrene of the Δ*uge3* strain pre-incubated for 30 min with a suspension of GAG or zymosan at the indicated concentrations. (E) Scanning electron micrographs of hyphae of the indicated strains after 24 h of growth and co-incubation with a suspension of GAG or zymozan for 30 min, when indicated. Magnification was 20,000×. All assays were performed on at least three independent occasions. Graphs indicate mean ± standard error. * indicates a significantly reduced adherence of the Δ*uge3* mutant as compared with Af293 and *uge3*-complemented strains (B) or a significantly increased adherence of the *uge3* mutant after carbohydrate addition as compared with *uge3* mutant alone (C), *p*<0.05 by factor ANOVA.

**Figure 5:**
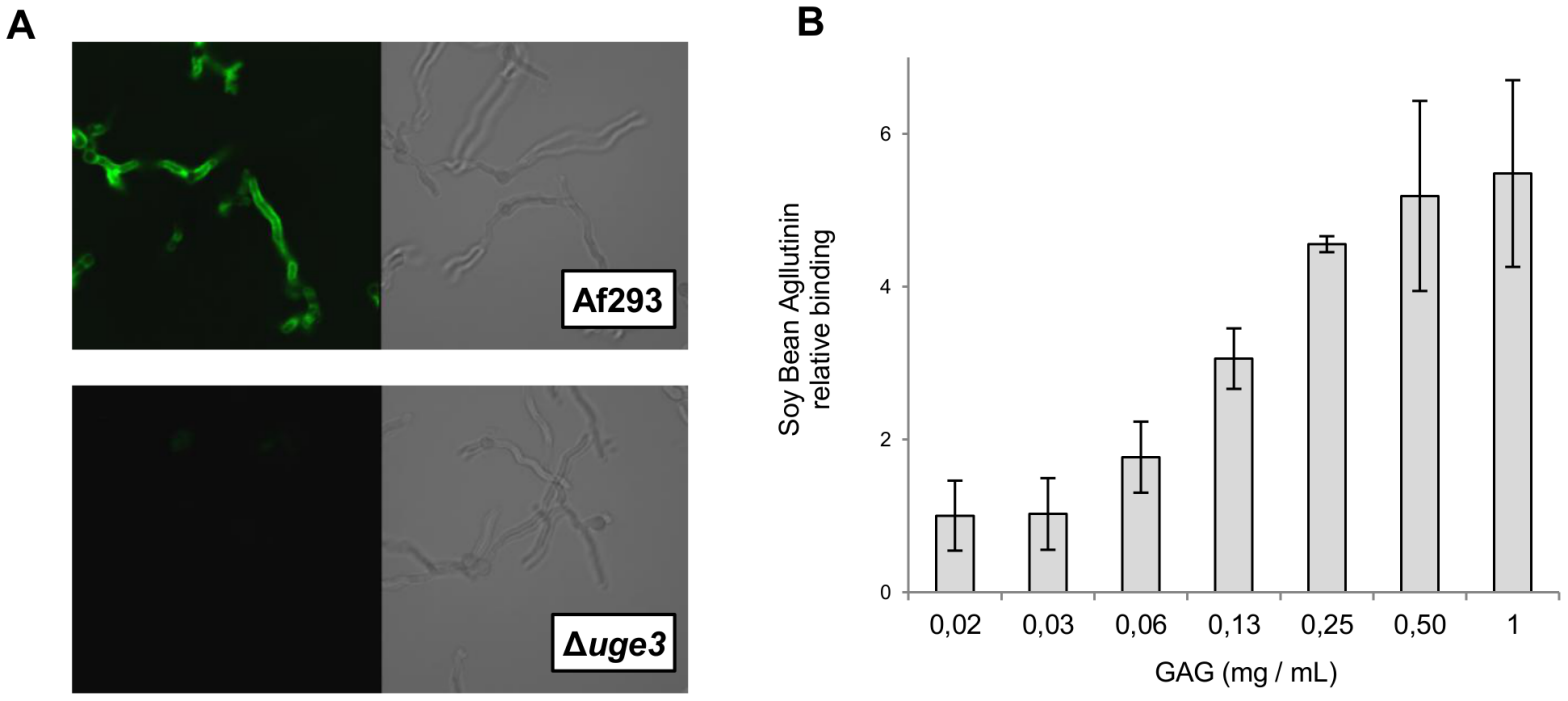
GAG binds to epithelial cells. (A) Soy Bean Agglutinin (SBA), a GalNAc binding lectin, detects GAG on hyphae. Hyphae were grown for 12 h, fixed, stained with FITC-conjugated SBA and imaged using confocal microscopy. Magnification was 40×. (B) Dose dependent binding of purified GAG particles to A549 cells. Cells were co-incubated with the indicated concentration of GAG, and then washed and bound GAG was quantified by detection with FITC-conjugated SBA. The assay was performed on three independent occasions. Graphs indicate mean ± standard error.

To test the hypothesis that GAG was required to mediate *A. fumigatus* adherence to substrates, we compared the adherence of the Δ*uge3* mutant with the wild-type and *uge3* complemented strains to a variety of substrates. The Δ*uge3* mutant strain exhibited a near complete absence of adherence to all substrates tested, including plastic, pulmonary epithelial cells and fibronectin (Fig. 4A, 4B). As it was observed with the Δ*stuA* and Δ*medA* mutant strains, preincubation of either plastic plates or hyphae of the Δ*uge3* mutant with a suspension of wild-type GAG produced a dose dependent increase in adherence to plastic (Fig. 4C, 4D). This increased adherence was not observed when the wells or hyphae were supplemented with a suspension of zymosan, a β-glucan-rich fungal cell wall preparation, suggesting that the increased adherence is specific to GAG. Scanning electron microscopy of Δ*uge3* hyphae incubated with a suspension of GAG demonstrated a partial restoration of the cell wall decoration seen in wild-type hyphae, suggesting that the Δ*uge3* mutant was able to bind extracellular GAG (Fig. 4E). To confirm that GAG directly binds to epithelial cells, we tested the ability of a suspension of GAG isolated from wild-type *A.fumigatus* to bind directly to A549 cells. FITC conjugated Soy Bean Agglutinin (SBA) was used to quantify GAG binding. This lectin is specific for terminal GalNAc residues, and does not bind to GAG deficient uge3 mutant hyphae (Fig. 5A). Using this approach, purified GAG was observed to bind to A549 epithelial cells in a dose dependent manner (Fig. 5B). Collectively these results demonstrate that GAG is required for adherence to, and injury of epithelial cells, and suggest that GAG is an important adhesin of *A. fumigatus*.

**Figure 6:**
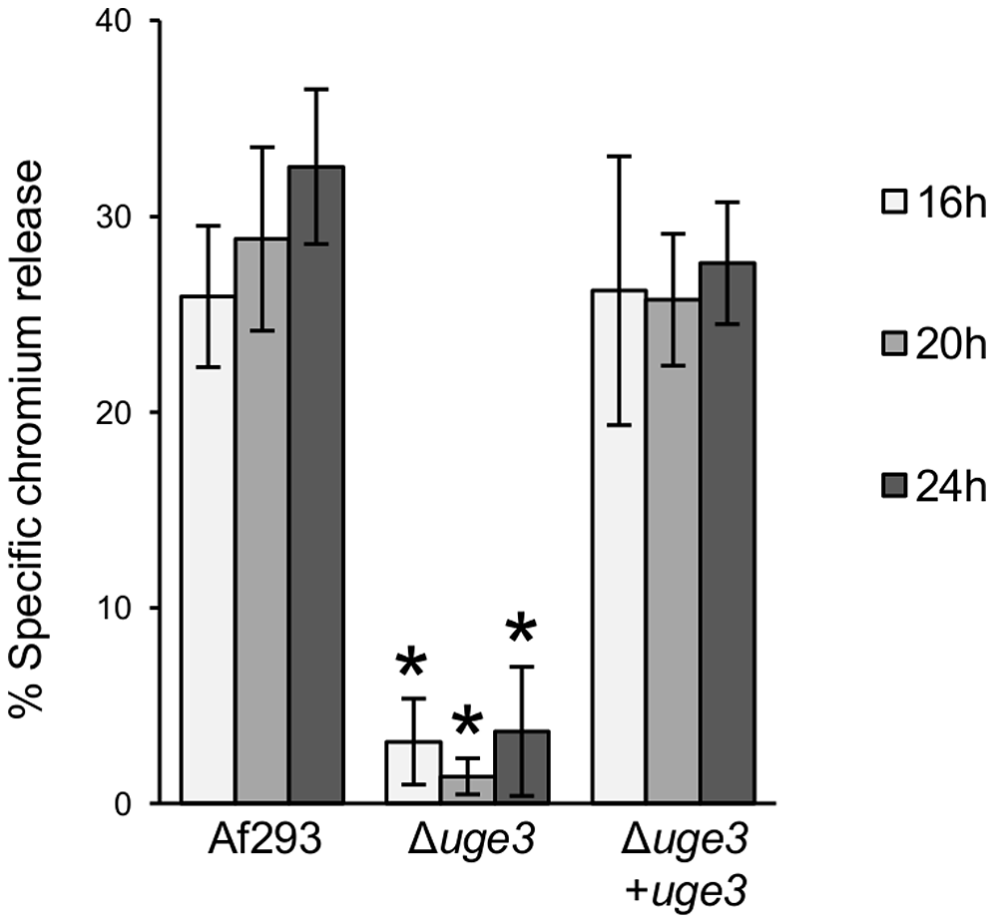
Uge3 is required for the induction of epithelial cell injury by *A. fumigatus*. A549 pulmonary epithelial cells were incubated with conidia of the indicated strains for 16, 20 or 24 h, after which the extent of epithelial cell injury was measured with a chromium release assay. Graphs indicate mean ± standard error of 3 independent experiments, each performed in triplicate. * indicates a significantly reduced injury to cells induced by the Δ*uge3* mutant, as compared with injury induced by Af293 and *uge3*-complemented strains, *p*<0.05 by factor ANOVA.

Deletion of *uge3* also completely blocked the ability of *A. fumigatus* to induce pulmonary epithelial cell injury as measured by a chromium release assay (Fig. 6). Restoration of *uge3* expression in the Δ*uge3* mutant completely restored the ability of *A. fumigatus* to adhere to host constituents and damage epithelial cells, confirming the specificity of these observations and suggesting that GAG is necessary for adherence to host constituents and subsequent induction of epithelial cell injury.

### The *uge3* deficient mutant is attenuated in virulence and induces an increased inflammatory response *in vivo*

**Figure 7:**
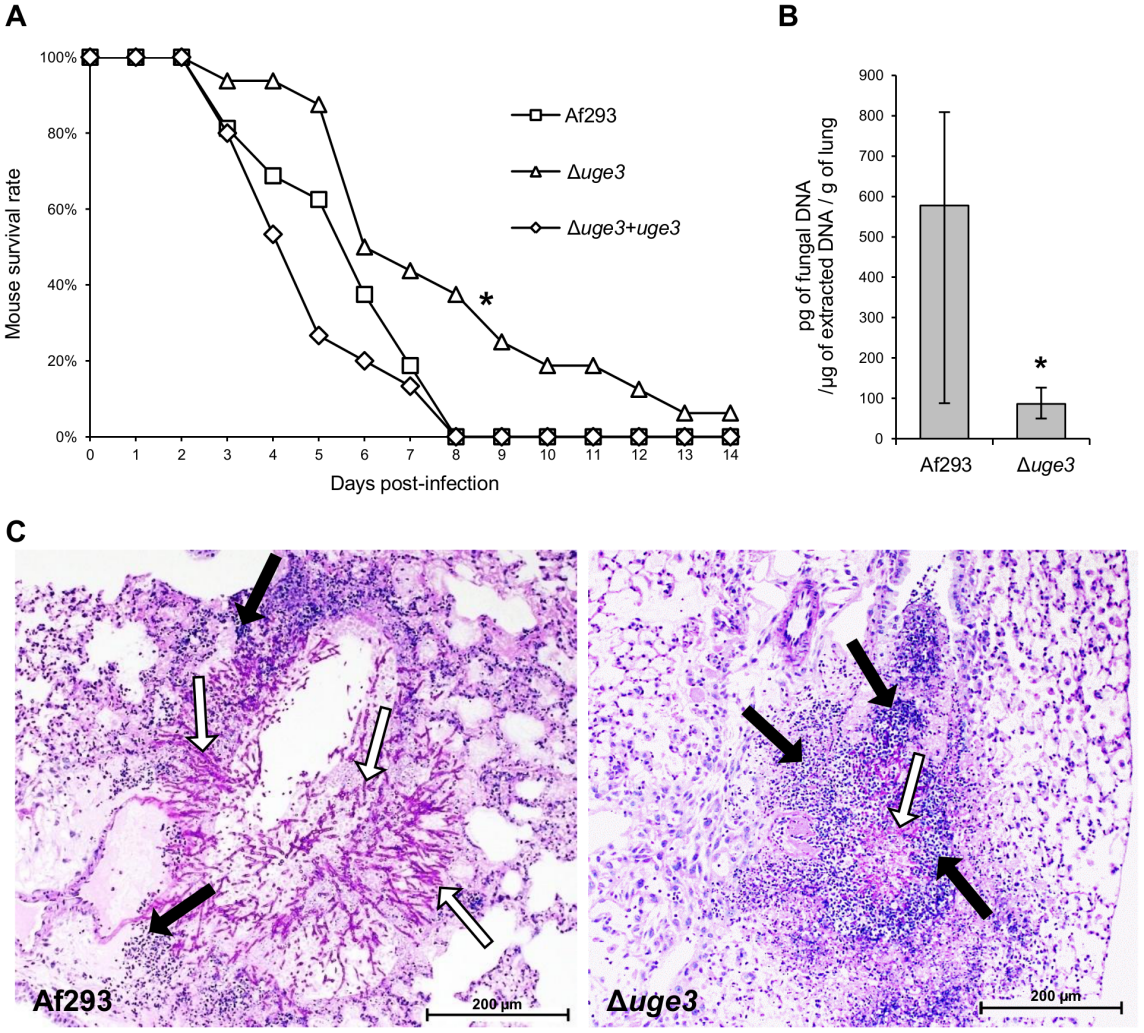
Uge3 is required for full virulence in mice model of IA. (A) Survival of cortisone acetate-treated Balb/C mice infected with the indicated *A. fumigatus* strains. Data are the combined results of 2 independent experiments for a total of 16 mice per strain. * indicates a significant increase in survival between mice infected with the Δ*uge3* mutant and mice infected with the wild-type or with the *uge3* complemented strain, *p* = 0.017 and <0.001 respectively by the log rank test. (B) Quantification of fungal DNA in lung homogenates of mice after 4 days of infection. Results are median ± interquartile of 8 mice per strain. * indicates a significantly reduced fungal DNA content in lungs of mice infected with the Δ*uge3* mutant compared with those infected with the wild-type strain, *p* = 0.015 by the Wilcoxon rank sum test. (C) Photomicrographs of PAS stained sections of mouse lungs obtained 4 days after infection with the indicated strains. White arrows indicate hyphae, black arrows indicate infiltrating leukocytes.

To determine if blocking GAG synthesis and fungal adherence alters virulence, we compared the virulence of the Δ*uge3*, wild-type *A. fumigatus* and the *uge3* complemented strain in a corticosteroid treated mouse model of invasive aspergillosis. Mice infected with the Δ*uge3* mutant strain survived significantly longer than mice infected with either the wild type of *uge3*-complemented strain (Fig. 7A), although this effect was modest. Consistent with the increased survival of mice infected with the Δ*uge3* mutant, these mice were found to have a significantly reduced pulmonary fungal burden after four days of infection as compared with mice infected with wild-type *A. fumigatus* (Fig. 7B). Histopathologic examination confirmed that infection with the Δ*uge3* mutant strain produced fewer and much smaller fungal lesions than did the wild-type *A. fumigatus* (Fig. 7C). Surprisingly, despite the lower abundance of hyphae in pulmonary lesions of mice infected with the Δ*uge3* mutant, these lesions contained more neutrophils than did those of mice infected with wild-type *A. fumigatus*. These results suggest that infection with the Δ*uge3* mutant strain induced an increased host inflammatory response.

### Galactosaminogalactan masks β-glucan exposure on the surface of *A. fumigatus* during hyphal growth

**Figure 8:**
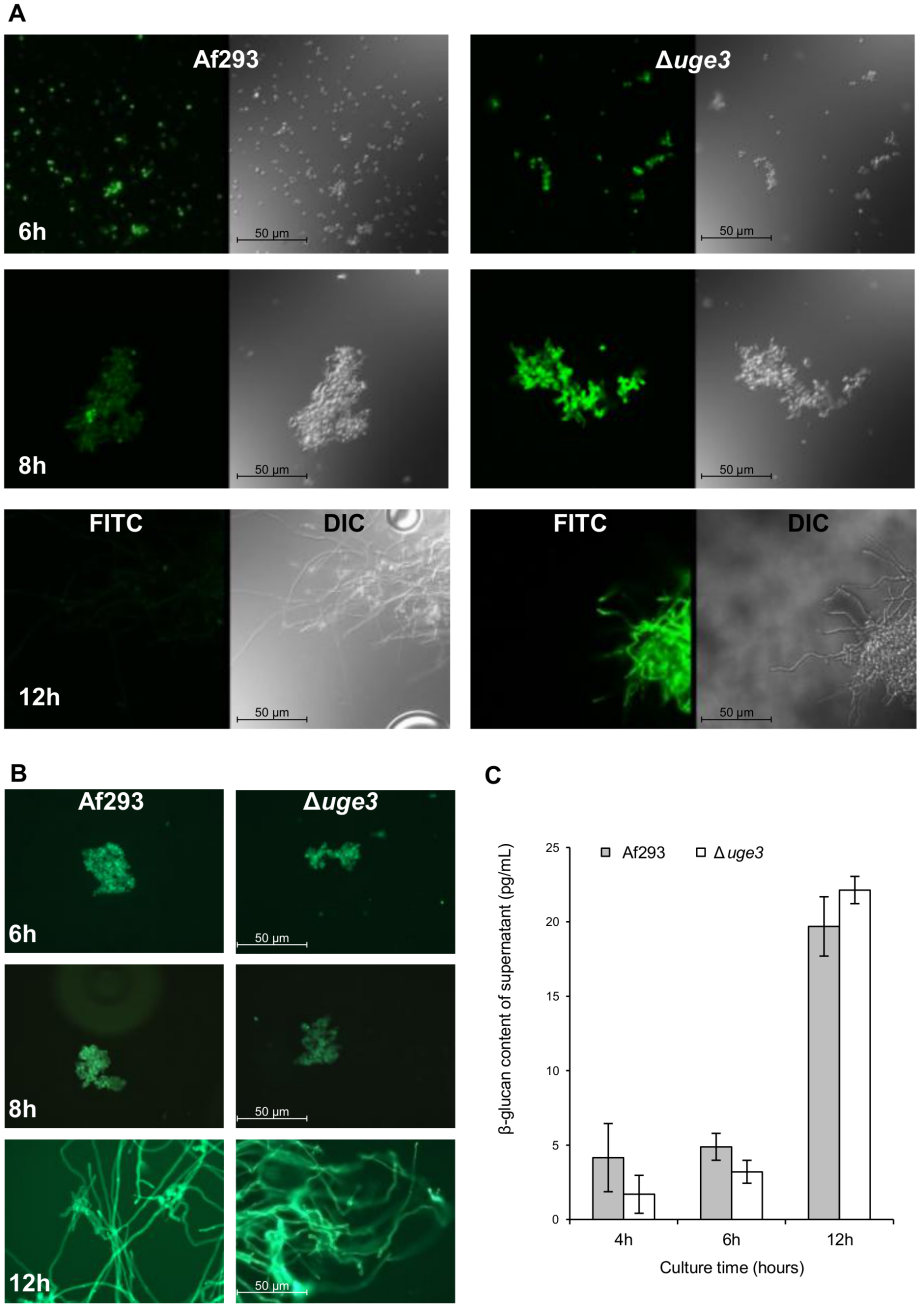
β-glucans are exposed on hyphae in the absence of GAG. Detection of Fc-dectin-1 binding using indirect immunofluorescence. Conidia of *A. fumigatus* strains Af293 and Δ*uge3* were grown in Brian media for the indicated times, to produce swollen conidia (6 h), early germinating conidia (8 h) or hyphae (12 h). Samples were then fixed and stained with Fc-dectin-1 (A) or with aniline blue (B) and imaged with epifluorescent microscopy at 40× magnification. (C) β-glucan content of supernatants harvested from the indicated strains, as determined by the (1→3)-β-D-Glucan Detection Reagent Kit. Results are the mean ± standard error of two independent experiments.

GAG has been localized to the amorphous outer layer of the fungal cell wall [15]. We therefore hypothesized that extracellular GAG might mask the surface exposure of other fungal pathogen-associated molecular pattern (PAMP) molecules such as β-1,3 glucan, and as a result the increased inflammatory response seen during infection with the Δ*uge3* mutant might be a consequence of unmasking of these PAMPs. To test this hypothesis, we performed immunofluorescent microscopy to compare the binding of recombinant Fc-dectin-1 [17] to the Δ*uge3* mutant and wild-type *A. fumigatus*. Consistent with previous reports [18], we found that binding of Fc-dectin-1 to swollen conidia could be detected in both strains (Fig. 8A). However, during germination and hyphal growth, there was much more intense staining of Δ*uge3* mutant hyphae as compared with the wild-type parent strain, in which Fc-dectin-1 binding decreased over time. In contrast, total β-1,3 glucan content, as assessed by aniline blue staining and release of soluble β-1,3 glucan in the culture supernatant, was not different between the wild-type and the Δ*uge3* mutant strains (Fig. 8B, 8C). Similarly, sensitivity of the Δ*uge3* mutant to the β-1,3 glucan synthase inhibitor casofungin was unchanged from the wild-type parent strain (Table 1). Therefore, the increased binding of Fc-dectin-1 to the Δ*uge3* cells was due to greater surface exposure of β-1,3 glucan rather than increased synthesis of this glycan.

### GAG deficient strains induce a dectin-1 dependent increase production of pro-inflammatory cytokines by dendritic cells *in vitro*

**Figure 9:**
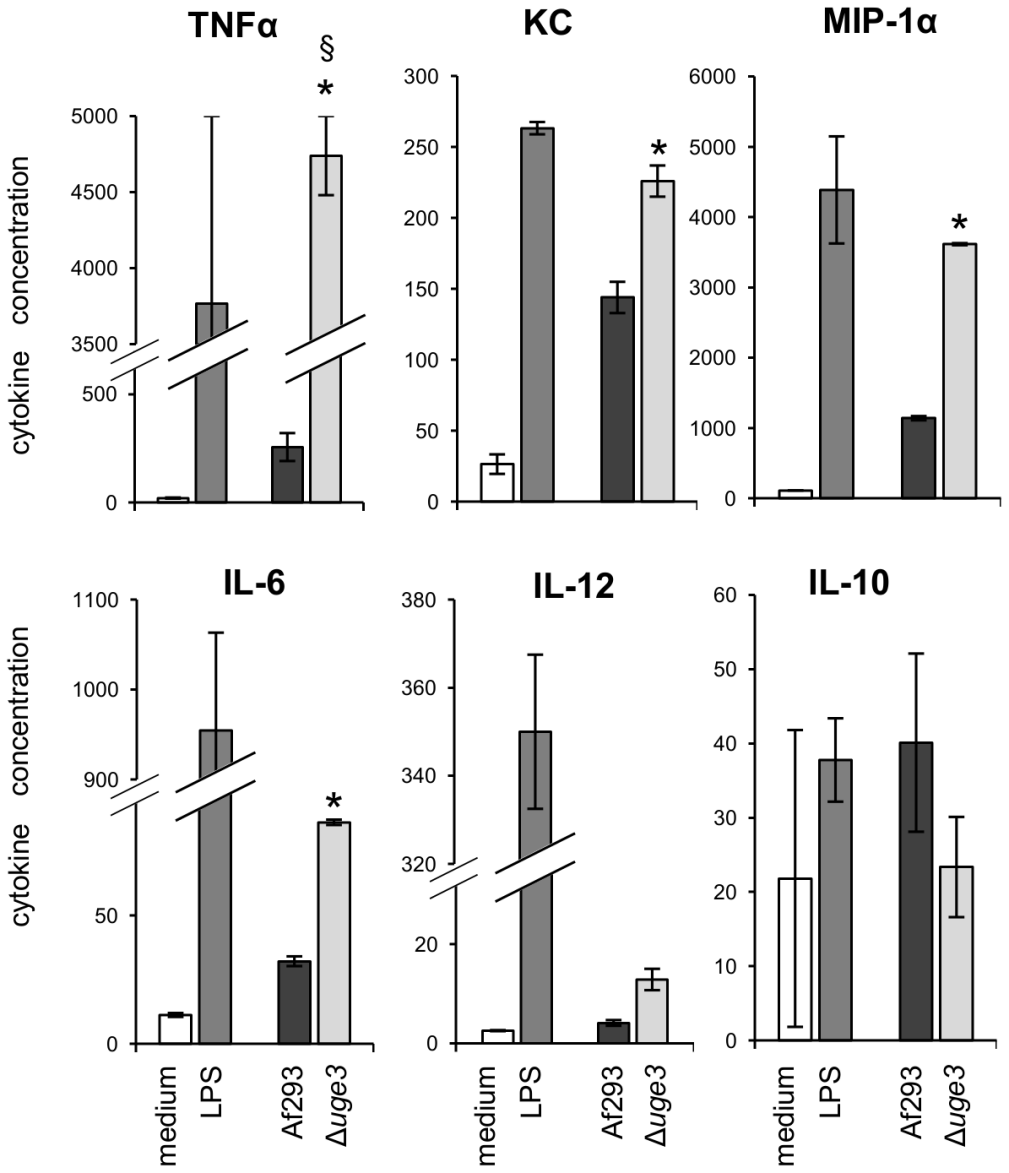
Dendritic cells produce an increased pro-inflammatory cytokine profile in response to the GAG deficient mutant. Graphs show the cytokine content of culture supernatant after 6 h of infection of BMDDCs with hyphae of the indicated strains. LPS was used as a positive control, and medium as a negative control. Cytokine concentrations in culture supernatants were determined by multiplex EIA. Results are mean ± standard error of duplicate determination of cytokine concentrations, indicated in pg/mL. * indicates a significantly increased cytokine concentration induced by the Δ*uge3* mutant, as compared with the one induced by Af293, *p*<0.05 by factor ANOVA. § indicates that actual value is above 5,000 pg TNFα/mL (measures exceeded upper limit of the test).

To test if the increased exposure of β-glucan, or other fungal cell wall PAMPs, on the surface of Δ*uge3* hyphae might induce an increased inflammatory response by immune cells, we determined the cytokine response of bone marrow derived dendritic cells (BMDDCs) upon co-culture with wild-type or Δ*uge3* hyphae. After 6 hours of co-incubation, BMDDCs infected with the Δ*uge3* mutant strain produced significantly higher levels of pro-inflammatory cytokines, including TNF-α, KC, MIP-1α, IL-6, and a trend to higher IL-12 levels, as compared to BMDDCs infected with the wild-type strain (Fig. 9). In addition, a trend to lower levels of the anti-inflammatory cytokine IL-10 produced by BMDDCs infected with the Δ*uge3* mutant as compared with hyphae of wild-type *A. fumigatus* was observed, although this difference was not statistically significant.

**Figure 10:**
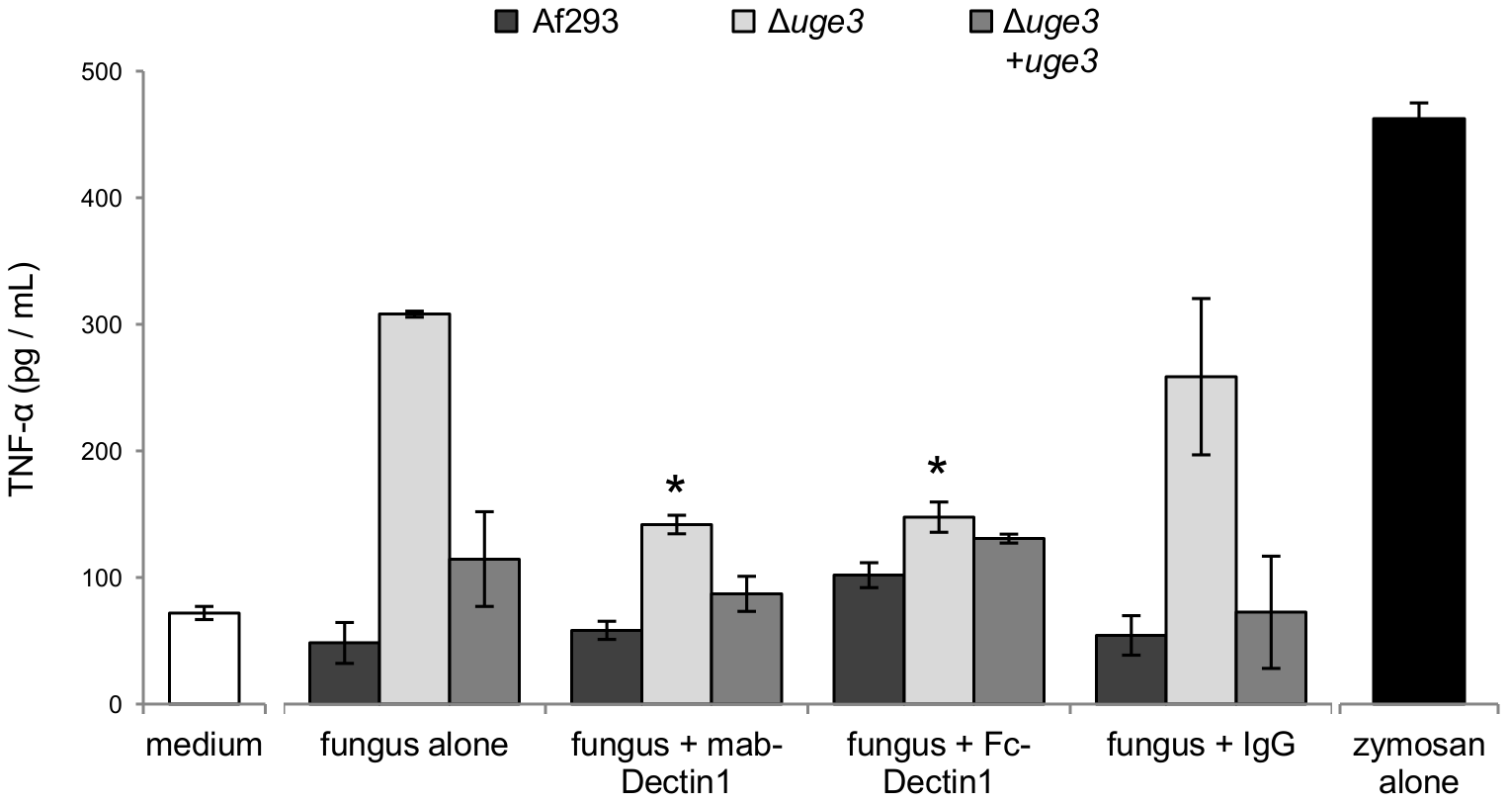
Dectin-1 blockade abrogates the increased TNFα secretion induced by the GAG deficient Δ*uge3* mutant. BMDDCs were infected with hyphae of the indicated strains for 6 h, after which the TNFα content of culture supernatants was determined by EIA. Dectin-1 recognition of β-glucan was inhibited by preincubating BMDDCs with a monoclonal anti-dectin 1 antibody or by preincubating hyphae with Fc-dectin-1. Results are mean ± SEM of duplicate experiments, each performed in triplicate. * indicates a significant reduction of TNFα production compared to BMDDCs exposed to the Δ*uge3* mutant without dectin-1 blocking, *p*<0.05 by factor ANOVA.

To confirm these results and determine if this increased pro-inflammatory response induced by the Δ*uge3* mutant was mediated by increased binding to dectin-1, we examined the ability of an anti-dectin-1 neutralizing antibody and Fc-dectin-1 to block the increase in TNF-α production by BMDDCs in response to hyphae of the Δ*uge3* mutant strain. Pre-incubation of BMDDCs with a monoclonal anti-dectin-1 antibody completely blocked the increased TNF-α production by BMDDCs in response to hyphae of the Δ*uge3* mutant strain (Fig. 10). Similarly, pre-incubating hyphae of the Δ*uge3* mutant strain with Fc-dectin-1 completely blocked the increased TNF-α production by BMDDCs. Collectively these results support the hypothesis that GAG inhibits host inflammatory responses in part by masking of PAMPs such as β-glucan.

### GAG is essential for virulence in highly immunocompromised mice

**Figure 11:**
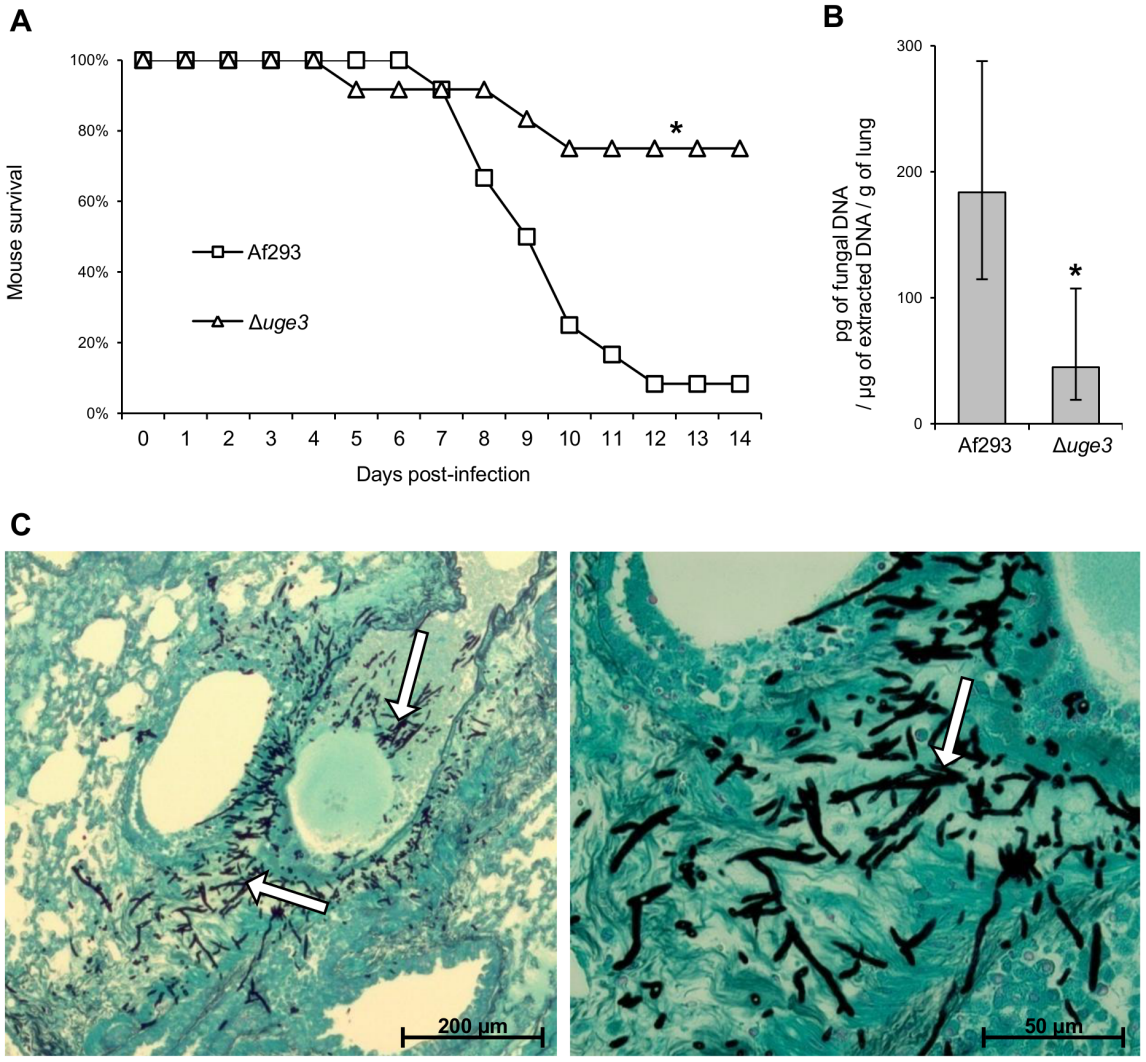
Uge3 is required for full virulence in a highly immunosuppressed mouse model of IA. (A) Survival of highly immunosuppressed mice treated with cyclophosphamide and cortisone acetate and infected with the indicated *A. fumigatus* strains. * indicates significantly increased survival of mice infected with the Δ*uge3* mutant as compared with those infected with the wild-type, *p* = 0.002 by the log rank test (*n* = 12 mice per fungal strain). (B) Quantification of fungal DNA in lung homogenates of mice after 5 days of infection. Results are median ± interquartile range of 8 mice per strain. * indicates a significantly reduced fungal DNA content in lungs of mice infected with the Δ*uge3* mutant as compared with those infected with the wild-type strain, *p* = 0.11 by the Wilcoxon rank sum test. (C) Photomicrographs of Gomori menthenamine silver stained sections of mouse lungs obtained 4 days after infection with the wild-type strain Af293. No fungal lesions could be identified in the lungs of mice infected with the Δ*uge3* mutant strain. Magnification was ×100 and ×400. White arrows indicate hyphae. Note the lack of infiltrating leukocytes within fungal lesions.

The results of these *in vitro* and *in vivo* studies suggest that the unmasking of fungal PAMPs in the absence of GAG induces an increased inflammatory response to hyphae that is detrimental to the host. To test this hypothesis, the virulence of the Δ*uge3* mutant and wild-type strain was compared for their virulence in highly immunosuppressed mice treated with both corticosteroids and cyclophosphamide. In this model, *A. fumigatus* infection does not induce a detectable cellular or cytokine inflammatory response during the neutropenic period [19]. In these highly immunosuppressed mice, the Δ*uge3* mutant strain exhibited markedly attenuated virulence as compared with the wild-type parent strain (Fig. 11A). This difference in virulence was unlikely related to differences in the initial infectious inoculum, since the fungal burden was similar between mice infected with the wild-type and Δ*uge3* mutant and sacrificed one hour after infection (a median of 1900 vs. 1850 colony forming units per animal, respectively). Mice infected with the Δ*uge3* mutant strain had a reduction in pulmonary fungal burden that was similar in magnitude to that seen in the non-neutropenic mouse model (Fig. 11B). Histopathologic examination of lungs after 5 days of infection confirmed an absence of infiltrating leukocytes surrounding the sites of wild-type *A. fumigatus* infection (Fig. 11C). These data suggest that the increased inflammatory response induced by the Δ*uge3* strain in non-neutropenic mice is non-protective and increases mortality, because inhibiting inflammation in the highly immunosuppressed mouse model was associated with improved survival.

**Figure 12:**
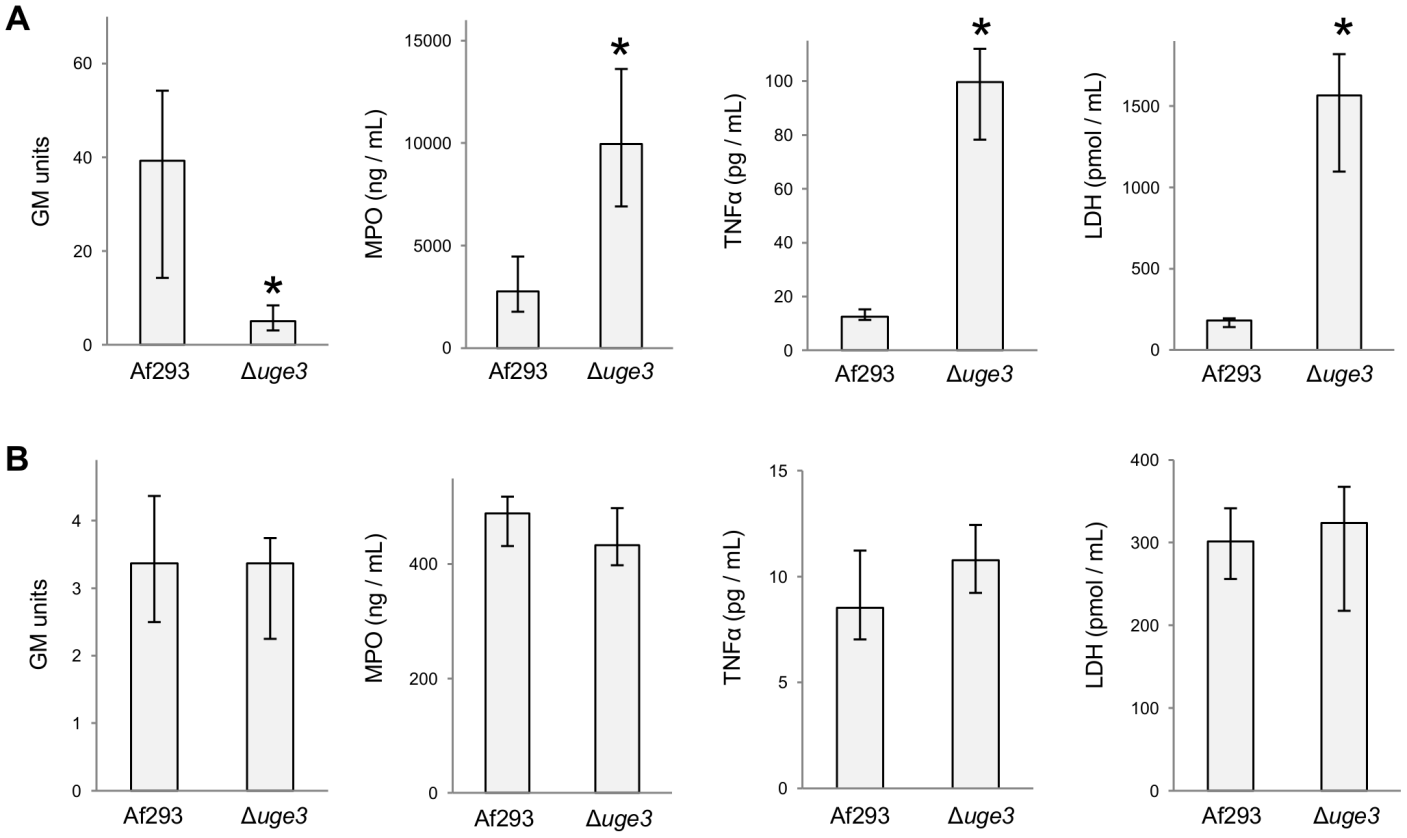
The *A. fumigatus* Δ*uge3* mutant induces a hyperinflammatory response in non-neutropenic mice that is attenuated in highly immunocompromised mice. (A.) Corticosteroid treated mice were infected by inhalation with the indicated strains of *A. fumigatus* and sacrificed three days after infection. Fungal burden was determined by pulmonary galactomannan content and pulmonary inflammation was measured by determining MPO, and TNF-α content. Pulmonary injury was quantified by measuring LDH release in BAL fluid. MPO, TNF-α, and LDH levels were normalized to the fungal burden of each strain in Panel 1. Results are median ± interquartile range of 8 mice per strain. * indicates a significant decrease in fungal burden or a significant increase in MPO, TNFα or LDH content in lungs of mice infected with the Δ*uge3* mutant as compared to the lungs of mice infected with the wild-type strain, *p*<0.01 by the Wilcoxon rank sum test. (B) Corticosteroid and cyclophosphamide treated mice were infected by inhalation with the indicated strains, sacrificed and the lungs processed as in (A). MPO, TNF-α, and LDH levels were normalized to the fungal burden of each strain in Panel 1. Results are median ± interquartile range of 9 mice per strain. Note: y-axis values for all graphs are lower than those in (A).

To confirm this hypothesis, we compared the inflammatory response during infection with the wild-type and the Δ*uge3* mutant in both the non-neutropenic model and the highly immunosuppressed models. To minimize the effects of differences in fungal burden between strains, mice were studied earlier in the course of disease, after three days of infection. In non-neutropenic immunosuppressed mice, a significantly lower fungal burden was again observed in mice infected with the Δ*uge3* mutant strain as compared with those infected with wild-type *A. fumigatus* (Fig. 12A). Relative to this lower fungal burden, Δ*uge3* mutant strain was found to induce significantly higher pulmonary myeloperoxidase levels (MPO) suggesting that this mutant has a higher capacity to mediate pulmonary leukocyte recruitment as compared with wild-type *A. fumigatus* (Fig. 12A). Similarly, the relative induction of pulmonary TNF-α, as well as the ability to induce pulmonary injury, as measured by LDH levels in BAL fluid, was significantly greater with the Δ*uge3* mutant than with wild-type *A. fumigatus* (Fig. 12A). In contrast, in the highly immunosuppressed mouse model there was no significant difference in pulmonary fungal burden, myeloperoxidase content, TNF-α levels or LDH release between mice infected with the wild-type and with the Δ*uge3* mutant strain at this earlier time point (Fig. 12B). Further, these measures of inflammation were significantly lower in these highly immunosuppressed mice as compared with non-neutropenic animals. Collectively these data suggest that in non-neutropenic mice, infection with the Δ*uge3* mutant stimulates a non-protective hyper-inflammatory response.

## Discussion

In *A. fumigatus*, GAG is a heterogeneous linear polymer consisting of α1–4 linked galactose and N-acetylgalactosamine residues in variable combination [16]. GAG is secreted and also a component of both the amorphous cell wall and extracellular matrix. In addition, GAG has been detected in lung lesions of experimentally infected animals [15]. The present study adds significantly to our understanding of the biosynthesis and function of this fungal polysaccharide.

First, the results of our *in vitro* studies strongly suggest that GAG is the principal mediator of *A. fumigatus* adherence and plays a key role in biofilm formation. The mechanism by which this carbohydrate mediates adherence to substrates and binds to hyphae remains undefined. Although specific host or fungal lectins may mediate binding of *Aspergillus* GAG, binding to plastic is clearly independent of host receptors and must be mediated by physicochemical interactions such as charge or hydrophobicity. Further, the lack of competition observed when GAG in suspension was added to wild-type hyphae would argue against a receptor-ligand interaction. Overall, these data are most consistent with a model in which GAG functions as a glue that mediates attachment between hyphae and substrates in a highly promiscuous fashion. The findings of this study add to the growing body of evidence implicating polyhexosamine glycans as key adhesion factors for microorganisms. Work from the 1970's identified polygalactosamine compounds from *Neurospora crassa* and *Bipolaris sorokiniana* and suggested that they could potentially play a role in the adherence of fungal spores to glass surfaces 20,21. Similarly, a large number of gram positive and gram negative bacterial biofilms contain polysaccharide intercellular adhesin (PIA), a homopolymer of N-acetylglucosamine, which mediates adherence between bacteria and the surfaces they colonize [22]. Although composed of a different amine sugar, the similarities between these mechanisms of adherence are striking. The adhesive characteristics of PIA are in large part governed by de-acetylation of N-acetyl glucosamine residues. PIA differs from *A. fumigatus* GAG in which the galactosamine residues have been reported to be uniformly acetylated [16]. Nevertheless, these data suggest that the use of polyhexosamine glycans is a widespread microbial adherence strategy, and could potentially serve as a useful target for the development of antimicrobial strategies with broad applicability.

The present results suggest that Uge3 is a key enzyme in the GAG biosynthetic pathway. The absence of N-acetyl galactosamine in the cell wall of the *uge3* mutant strain and the absence of effects on galactofuranose synthesis suggest that this enzyme functions in the production of N-acetyl galactosamine, although experimental validation of this hypothesis is required. A minimal increase in the GlcNAc content of the cell wall of the Δ*uge3* mutant was also noted. Although this finding could suggest accumulation of substrate in the absence of conversion to GalNAc, it is unclear if this is a significant finding. A similar increase in GlcNAc content was also seen in the *uge3* complemented strain despite a restoration of GalNAc and GAG synthesis. Similarly, we observed no difference in susceptibility to classic cell wall perturbing agents between the Δ*uge3* mutant and complemented strains, suggesting that the increased GlcNAC seen in both strains does not contribute to the marked reduction of adherence and virulence that was seen only in the Δ*uge3* mutant. Although these data suggests Uge3 therefore mediates synthesis of the N-acetyl galactosamine component of GAG, the pathways responsible for the production of galactose for the synthesis of GAG remain unknown. It is possible that Uge3 also mediates the interconversion of UDP-glucose to UDP-galactose, as epimerases with dual substrate affinity have been described [23], however the normal levels of galactose in the Δ*uge3* mutant strain argue against this hypothesis. Alternately, galactose synthesis may be dependent on one of the other two putative epimerases identified within the *A. fumigatus* genome.

The results of this and previous studies strongly suggest that GAG modulates immune responses in vivo. Previous work has suggested that GAG may be recognized by the host as a PAMP and mediate immunosuppression. Fontaine *et. al.* observed that a urea-soluble fraction of GAG induced neutrophil apoptosis in vitro, and that vaccination of mice with a soluble fraction of GAG enhanced the progression of invasive aspergillosis in immunocompetent and immunosuppressed mice in association with increasing Th2 and Th17 responses [16]. The experiments described here add substantially to these findings by testing the effects of live organisms deficient in GAG production in both non-neutropenic and highly immunosuppressed leukopenic mice. Our findings of increased local inflammation surrounding the Δ*uge3* strain support the role of GAG as an immunosuppressive molecule. The increased inflammatory response to GAG-deficient hyphae was not protective, but rather attenuated the survival advantage in mice infected with this strain when compared with highly immunosuppressed animals. These findings add support to the growing body of literature suggesting that non-protective inflammatory responses can increase mortality during infection with *A. fumigatus* [24], [25], [26].

In addition to its direct effects on the immune system, GAG likely also modulates host immune responses through cloaking β-glucan and possibly other PAMPs on the surface of hyphae. Masking of β-glucan and other cell wall PAMPs by the hydrophobin RodA has been previously demonstrated in conidia, and is thought to play an important role in immune evasion [27]. These cell wall β-glucans and other PAMPs are then exposed when the hydrophobin layer is shed during germination. However, studies examining β-glucan exposure during the growth and development of *A. fumigatus* hyphae have found that, as hyphae mature, the recognition of β-glucan exposure by dectin-1 decreases when compared with swollen conidia and early germinated hyphae [18]. Our results suggest that the production of GAG by maturing hyphae may account for this reduced exposure of β-glucan, and as in the case of conidia, results in an attenuation of inflammatory responses. A similar immune evasion strategy has been reported in the dimorphic fungus *Histoplasma capsulatum*, in which surface expression of α-(1,3)-glucan has been shown to mask exposure of β-glucan and reduce inflammatory responses [28]. This modulation of β-glucan exposure is the natural converse to the effects of the echinocandin antifungals, in which increasing the exposure of β-glucan is postulated to increase host inflammatory responses and improve fungal killing [29], [30].

The findings of this study also suggest that GAG-mediated adherence may play a role in virulence. Suppression of inflammation in mice infected with the Δ*uge3* mutant resulted in a reduced pulmonary fungal burden and increased survival of the mice infected with the Δ*uge3* mutant as compared to mice infected with the wild-type *A. fumigatus*. It is therefore possible that this attenuated virulence and reduced fungal burden reflects the impaired ability of this mutant to adhere to, and form colonies in the lung, rather than alterations in immune mediated fungal killing. Alternately, loss of GAG may render hyphae more susceptible to host killing by microbicidal peptide or other neutrophil-independent host defences, or result in a unique growth defect seen under in vivo conditions.

These studies suggest that anti-GAG strategies could be useful in the therapy of invasive aspergillosis. Importantly, however, our data would suggest that blocking GAG function would likely be a superior approach to inhibiting the synthesis of GAG, in order to avoid potentially increasing the inflammatory response, and potentially mortality, attributable to unmasking β-glucan or other PAMPs.

## Materials and Methods

### Fungal strains and growth conditions

*A. fumigatus* strain Af293 (a generous gift from P. Magee, University of Minnesota, St. Paul, MN) was used as the wild-type strain for all molecular manipulations. The Δ*medA*, Δ*ugm1* and Δ*stuA* mutants and their corresponding parent and complemented strains were described previously [10]. Except where indicated, strains were propagated on YPD agar and at 37°C while exposed to light as previously described [9]. Liquid growth media were synthetic Brian medium [31], Aspergillus Minimum Medium (AspMM) [32], and RPMI 1640 medium (Sigma-Aldrich) buffered with 34.53 g of MOPS (3-(N-morpholino)propanesulfonic acid, Sigma-Aldrich) per liter, pH 7.0 as indicated. When noted, pH and/or iron concentration were modified in AspMM, in order to generate a pH from 4.5 to 8.5, and a [Fe^2+^] from 0 to 30 µM. Microaerophilic growth was performed using YPD or AspMM, incubated in a candle jar.

### Tissue culture

The type II pneumocyte cell line CCL-185 (lung epithelial cells A549) was obtained from the American Type Culture Collection, and was grown in DF12K medium containing 10% foetal bovine serum, streptomycin (100 mg/litre) and penicillin (16 mg/litre) (Wisent).

Bone marrow derived dendritic cells (BMDDCs) were prepared by flushing femurs and tibias of 6–8 week old C57BL/6 mice. Bone marrow cells were then cultured with culture media supplemented with either J558L culture supernatants or rGM-CSF, as previously described [33], [34]. Marrow cells were plated at a density of 4×10^5^ cells/ml in petri dishes containing 10 ml of culture media. For J558L supernatant-supplemented cultures, on days 3 and 6, cells were fed an additional 1 ml J558L culture supernatant per dish, and on day 8 with 4 ml of culture media with 30% J558L culture supernatant per dish. BMDDCs were used in fungal interaction experiments after 11 days of culture. For rmGM-CSF-supplemented cultures, on day 3 marrow cells were fed an additional 4 ml of culture media, and used in experiments on day 9. BMDDC differentiation was confirmed by flow cytometry via CD11c expression (data not shown).

### Molecular and genetic manipulations

#### Mutant transcriptome analysis

RNA expression analysis of the Δ*medA* mutant was compared to the wild-type and *medA* complemented strains using an *A. fumigatus* Af293 amplicon microarray as described previously [10], [14]. To obtain RNA for microarray analysis, RPMI 1640 medium buffered with 4-morpholinepropanesulfonicacid (MOPS) to pH 7.0 (Sigma-Aldrich) was inoculated with conidia of the various strains at a final concentration of 10^6^ conidia per ml. RNA was harvested after 8, 18, and 24 h, as described above. An *A. fumigatus* Af293 DNA amplicon microarray containing triplicate probes for 9516 genes was used in this study [35]. The labeling reactions with RNA and hybridizations were performed as described in the J. Craig Venter Institute standard operating procedure (http://pfgrc.jcvi.org/index.php/microarray /protocols.html). cDNA from the Δ*medA* mutant was hybridized against cDNA from the wild-type strain in three biological replicates and against cDNA from the Δ*medA*::*medA* complemented strain in two biological replicates. Dye swaps were performed. The gene expression ratios were log_2_-transformed and imported into JCVI MultiExperiment Viewer (MeV) software (http://www.tm4.org/mev.html) [36]. The Significance Analysis for Microarrays (SAM) method [37] was used (false discovery rate of 0.1%) to determine genes subject to differential transcriptional regulation between the control strains and the Δ*medA* mutant after 18 h and/or 24 h of growth. The results were then compared to the previously published results obtained in the same conditions with the set of strains Af293, Δ*stuA*, and Δ*stuA*::*stuA* [10].

#### Disruption of *uge3*

Our standard disruption protocol [10], [32] was adapted to the Gateway® (Invitrogen) system as follows: first plasmid pAN7.1 was modified for Gateway® use by digestion with *BmgBI* or *NaeI* followed by fusion of an *attR*::*ccdB* target sequence at the site of each digestion using the Gateway® Vector Conversion system, to generate plasmids pHY, and pYG. To generate the disruption constructs, ∼1 kb of the flanking sequences of *uge3* was amplified by PCR from Af293 genomic DNA using primers U1,U2 and U3,U4 to generate fragments FS1 and FS4 respectively (Table S1). The resulting PCR products were then cloned into pENTR-D-TOPO® entry plasmid. A LR recombination allowed recombination of pENTR::FS1 with pHY, and of pENTR::FS4 with pYG, resulting in the fusion of each flanking sequence with the *hph* cassette in plasmids pHY and pYG plasmids. Finally, the DNA fragments for transformation were generated by PCR, using the primers U1,HY with pHY::FS1 and U4,YG with pYG::FS4. Protoplasts of *A. fumigatus* Af293 were then transformed with 5 µg of each DNA fragment, as previously described [32]. Transformants were selected on 0.025% hygromycin enriched plates. Complete deletion of the *uge3* open reading frame was confirmed by PCR using primers U-ext1, U-ext4, U-RT sense,U-RT antisense, HY and YG (Table S1), by real-time RT-PCR to ensure a complete absence of *uge3* mRNA using primers U-RT sense and U-RT antisense (Table S1).

#### Construction of the *uge3*-complemented strain

To verify the specificity of the mutant phenotype, we constructed a complemented strain in which a wild-type copy of *uge3* was reintroduced in the Δ*uge3* strain, using a split marker approach [32]. Briefly, the phleomycin resistance (*ble* cassette) plasmid p402 was converted to the Gateway system as describe above using *XbaI* or *SacI* digestion and ligation of an *attR*::*ccdB* target sequence at the site of each digestion to generate plasmids pBL and pLE. A 2.4 kb DNA fragment containing the *uge3* ORF and 1 kb of upstream sequence was amplified by PCR from Af293 genomic DNA using primers U1,U5, and cloned into the Gateway pENTR-D-TOPO plasmid to generate pENTR::FS1-*uge3*. Next, the *uge3* cassette from pENTR::FS1-*uge3* was cloned upstream of the *ble* cassette in pBL by Gateway mediated LR recombination. Using the same approach, the *uge3* downtream flanking sequences from the previously constructed pENTR::FS4 were cloned downstream of the *ble* cassette in pLE. Finally, the DNA fragments for transformation were generated by PCR, using primers U1,BL for pBL::FS1-*uge3* and U4,LE for pLE::FS4. Protoplasts of the Δ*uge3* mutant were then transformed with 5 µg of each PCR product, as previously described [32]. Transformants were selected on 0.015% phleomycin enriched plates. Transformants were tested by PCR and by Southern blotting to ensure re-integration of *uge3*, and *uge3* expression was verified by real-time RT-PCR using primers U-RT sense and U-RT antisense (Table S1) to ensure that *uge3* mRNA production was restored.

### Real-Time RT-PCR

*In vitro*, expression of the genes of interest was quantified by relative real-time RT-PCR analysis as previously described [38]. The primers used for each gene are shown in Table S1. First strand synthesis was performed from total RNA with Quantitec Reverse Transcription kit (Qiagen) using random primers. Real-time PCR was then performed using an ABI 7000 thermocycler (Applied Biosystems) Amplification products were detected with Maxima® SYBR Green qPCR system (Fermentas) . Fungal gene expression was normalized to *A. fumigatus TEF1* expression, and relative expression was estimated using the formula 2^−ΔΔCt^, where ΔΔCt = [(Ct_target gene_)_sample_−(Ct*_TEF1_*)_sample_]/[(Ct_target gene_)_reference_−(Ct*_TEF1_*)_reference_]. To verify the absence of genomic DNA contamination, negative controls were used for each gene set in which reverse transcriptase was omitted from the mix.

### Fungal cell wall analysis

Cell wall extraction was performed as previously described [39]. Alkali soluble (AS) and alkali insoluble (AI) fractions were extracted as previously described [40]. Monosaccharides were determined by gas chromatography after hydrolysis, reduction and peracetylation of the AI and AS fractions [41] with *meso*-inositol as internal standard. Both hexose and hexosamine concentrations were expressed as percentages of the total cell wall.

#### β-glucan analysis

Total cell wall β-glucans were assayed by aniline blue staining [42]. Briefly, conidia were grown for 4, 6 or 12 hours at 10^5^ conidia/mL in YPD at 37°C and stained with 0.05% aniline blue (Sigma-Aldrich) in PBS buffered at pH 9.5. Stained cells were imaged with confocal microscopy (IX81, Olympus), excitation 400 nm and emission 455 nm.

Surface exposed β-glucans were assayed by immunostaining with an Fc-dectin-1 fusion construct (a generous gift from Dr G.D. Brown, University of Aberdeen) as described previously [18]. Briefly, fungal cells were fixed in 4% paraformaldehyde in PBS, and blocked in 3% bovine serum albumin supplemented with 0.2% sodium azide in PBS. Cells were then labelled with 10 µg/ml of Fc-dectin-1 [18] followed by FITC-labeled AffiniPure F(ab′) fragment donkey anti-human IgG, FCy fragment specific (Jackson ImmunoResearch). Stained cells were imaged with confocal microscopy (IX81, Olympus), excitation 495 nm and emission 519 nm.

Soluble β-glucan release was measured in culture supernatants using the Glucatell assay (Associates of Cape-Cod Inc.), following the manufacturer's instructions. Results were normalized to fungal biomass dry weight.

#### SEM

Hyphae were grown for 24 hours in phenol red-free RPMI 1640 medium on glass coverslips, fixed with 2.5% glutaraldehyde in 0.1 M sodium cacodylate buffer at 4°C overnight, sequentially dehydrated in ethanol, and critical-point dried. Samples were then sputter coated with Au-Pd and imaged with a field-emission scanning electron microscope (S-4700 FE-SEM, Hitachi).

#### Extracellular GAG and GM production

For both polysaccharide production assays, 50 mL of Brian medium was inoculated with 5×10^7^ conidia, and incubated for 24 h at 37°C. Culture supernatant aliquots were harvested by filtration of the culture on nylon membrane. Extracellular GAG was precipitated by 2.5 volume of ethanol overnight night at 4°C, washed with 60% ethanol, lyophilized and weighed as described previously [16]. The precipitate was homogenized in 40 mM HCl and sonicated. Next, the galactosamine content was analyzed by total acid hydrolysis (HCl 6.6 N, 100°C, 4 h) and quantified with HPAEC on a CarboPAC-PA1 column (4.6×250 mm, Dionex) using NaOH (18.8 mM) and sodium acetate (0.3 M) in 0.1 M NaOH as eluent A and B, respectively, as described [43]. In addition, absence of protein in GAG fractions was verified by Bradford assay. Extracellular GM culture supernatant content was assayed by EIA using the Platelia® *Aspergillus* kit (Bio-Rad), following the manufacturer instructions. Both assays were performed on 3 independent occasions.

### Adherence assays

The ability of strains to form biofilms was tested by inoculating 6- well culture plates with 10^5^ conidia in 1 mL of Brian broth. After incubation at 37°C for 24 h, the plates were washed, fixed and stained as previously described [9].

The capacity of the various strains of *A. fumigatus* to adhere to plastic, fibronectin and epithelial cells was analyzed using our previously described method [9]. Six-well culture plates were prepared with confluent monolayers of A549 epithelial cells, adsorbed with 0.01 mg/ml of fibronectin overnight, or left untreated and then infected with 200 germlings of the strain of interest in each well and incubated for 30 minutes. Following incubation, wells were washed 3 times with 4 mL of PBS in a standardized manner, and overlaid with YPD agar for quantitative culture. The adherence assays were performed in triplicate on at least three separate occasions.

For carbohydrate supplementation experiments, either the plastic plate or the germlings were coated with the carbohydrate of interest. Briefly, the appropriate amount of extracellular GAG isolated as above, or of zymosan (Sigma-Aldrich), was resuspended in 2 mL of PBS by sonication, added to the wells of a 6-well non-tissue culture plate and incubated overnight before being washed and used in the adherence assay described above. For adherence assay with glycan treated fungus, germlings were incubated for 1 hour, at room temperature, in a dilution of GAG or zymozan in PBS; then rinsed three times to remove non-adherent carbohydrate before being tested for adherence as described above.

To measure the adherence of purified GAG to A549 epithelial cells, monolayers of A549 cells were grown to confluence in 96-well plate (Nunclone, Inc) then fixed in 4% paraformaldehyde. Cells were incubated with varying concentrations of GAG suspended in PBS, washed and then stained with fluorescein conjugated Soybean Agglutinin (Vector Labs). Binding was quantified by measuring fluorescence at 495 nm using Spectramax (Molecular Devices). To confirm the specificity of Soybean Agglutinin (SBA) for GalNAc residues of GAG, hyphae of Af293 wild-type, Δ*uge3*, and Δ*uge3::uge3* were grown on poly-d-lysine coated glass coverslip for 12 hours, fixed with 4% paraformaldehyde, co-incubated with fluorescein conjugated SBA, and imaged by confocal microscope at 488 nm (Olympus).

### Antifungal drug susceptibility assays

Caspofungin (Merck) and nikkomycin X (Sigma-Aldrich) were diluted in sterile deionized H_2_O. Calcofluor white (Sigma-Aldrich) was diluted in a solution of 0.8% KOH and 83% glycerol. Antifungal susceptibility testing was performed in accordance with the CLSI M38-A document for broth dilution antifungal susceptibility testing of filamentous fungi [44] as previously described [45]. Final dilutions of antifungals were prepared in RPMI 1640 buffered with MOPS. 100 µL of drug stock was added to 100 µL of 10^5^ conidia/mL solution per well. Plates were examined after 24 and 48 hours of incubation and the minimal inhibitory concentration (MIC) was determined by visual and microscopic inspection resulting in 100% growth inhibition while the minimal effective concentration (MEC) was determined by visual and microscopic inspection resulting in abnormal growth.

### Epithelial cell damage assay

The extent of damage to epithelial cells caused by the various strains of *A. fumigatus* was determined using a minor modification of our previously described method [46]. Briefly, A549 cells were loaded with chromium by incubating monolayers grown in 24-well tissue culture plates with 3 µCi of ^51^Cr at 37°C in 5% CO_2_ for 24 hours. Excess chromium was removed by washing with HBSS. The labelled A549 cells were then infected with 5×10^5^ conidia in 1 ml serum free DF12K medium. After a 16 h incubation, the medium above the cells was retrieved. The cells were then lysed with 6 N NaOH and the lysate collected. The ^51^Cr content of the medium and lysates was then measured in a gamma counter and the extent of epithelial cell damage was calculated. Each strain was tested in triplicate on three separate occasions, and all results were corrected for spontaneous chromium release by uninfected epithelial cells.

### Dendritic cell stimulation with *A. fumigatus* strains

*A. fumigatus* conidia were germinated for 9 h in non-tissue culture treated six-well plates in 2 ml phenol red-free RPMI 1640 medium at a concentration of 1.5×10^6^ conidia/well and allowed to germinate. Next, 1.5×10^6^ BMDDCs were then added to each well in 1 ml of RPMI 1640 medium. As a positive control BMDDCs were incubated with 3 µg/ml lipopolysaccharide (purified from *S. minnesota*, Invitrogen). Following 6 hrs co-incubation, culture supernatants were collected. Total cytokine analysis in culture supernatants was performed using the Mouse Cytokine 20-Plex Panel (Invitrogen), as per manufacturer's instructions, and analyzed using xPONENT analysis software. To investigate neutralization of either dectin-1 or β-glucan, BMDDCs or fungi were co-incubated for 1 h with 10 µg/ml mouse anti-dectin-1 (Invivogen) or 10 µg/ml Fc-dectin-1 recombinant protein (a generous gift from G. D. Brown), respectively. BMDDCs were added to fungi at a MOI of 1∶2. As controls, BMDDC or 5 µg/ml zymosan (Sigma-Aldrich) were co-incubated with fungi or BMDDC, respectively. TNF-α analysis in culture supernatant was performed using the Mouse TNF alpha ELISA Ready-SET-Go kit (eBiosciences).

### Virulence studies

The virulence of the indicated *A. fumigatus* strains was tested in two different murine models of invasive pulmonary aspergillosis. In the first model, male BALB/C mice were immunosuppressed by administering 10 mg of cortisone acetate (Sigma-Aldrich) subcutaneously every other day, starting on day −4 relative to infection and finishing on day +4, for a total of 5 doses [47]. In the second model, the mice were immunosuppressed with cortisone acetate, 250 mg/kg subcutaneously on days −2 and +3, and cyclophosphamide (Western Medical Supply), 250 mg/kg intraperitoneally on day −2 and 200 mg/kg on day +3 [48], [49]. For each fungal strain tested, groups of 11–13 mice were infected using an aerosol chamber as previously described [48]. An additional 8 mice were immunosuppressed but not infected. To prevent bacterial infections, enrofloxacin was added to the drinking water while the mice were immunosuppressed. Mice were monitored for signs of illness and moribund animals were euthanized. All procedures involving mice were approved by the Los Angeles Biomedical Research Institute Animal Use and Care Committee, and followed the National Institutes of Health guidelines for animal housing and care. In both models, differences in survival between experimental groups were compared using the log-rank test.

#### Determination of fungal burden

11 mice per strain were immunosuppressed and infected with the strains of interest in a separate experiment. After 4 days of infection, the mice were sacrificed and their lungs were harvested. They were immediately homogenized in ice cold PBS containing 10 µL of protease inhibitor mix/mL (Sigma Aldrich), and then aliquoted and stored at −80°C until use. The fungal burden was estimated by the total concentration of fungal genomic DNA [50]. Briefly, total DNA was extracted from the lungs using High Pure PCR Template Preparation Kit (Roche diagnostics) following the manufacturer's instructions. Real-Time PCR quantification of fungal DNA was performed on 150 ng of total lung DNA, using TaqMan Universal PCR Master Mix (Roche diagnostics) with a pair of oligonucleotides and a FAM-labelled probe for the amplification and detection of the 18S rRNA gene (Table S1). qPCR run was as follow: 2 min at 50°C; 10 min at 95°C; 50 cycles of (15 sec at 95°C+1 min at 60°C). The concentration of fungal DNA in the total lung extracted DNA was determined from a standard curve of serially diluted genomic DNA from strain AF293 (from 20 pg to 2.5 ng of fungal DNA per qPCR well). Fungal DNA concentration was normalized to lung weight and total DNA yield. To avoid the healthy survivor bias, initial fungal burden studies were performed on the fourth day after infection at which point 90% of infected animals remained alive. Subsequent experiments were performed on the third day of infection to minimize differences in fungal burden between mice infected with the wild-type and Δ*uge3* mutant strains. In experiments in which inflammation was measured, pulmonary galactomannan (GM) content of lung homogenates was used as a surrogate measure of fungal burden using the Platelia *Aspergillus* EIA kit (Bio-Rad) as we have done previously [9], [12], [13], [51], in order to preserve the lung tissue for the assays below. All strains produced similar levels of galactomannan during growth in vitro. Lung homogenates were clarified by centrifugation, diluted 1∶20 and assayed as per manufacturer's recommendations. Samples were compared to a standard curve composed of serial dilutions of lung homogenate from a mouse heavily infected with strain Af293.

#### Characterization of pulmonary inflammation

Mice were sacrificed and the airway contents were recovered by instillation and retrieval of 1 ml of sterile PBS through a needle inserted in the trachea. A total of three lavages were performed and pooled. LDH determination in BAL fluid was performed using a commercial assay (Abcam) following the manufacturer's instructions. 50 µl of each sample was assayed without dilution. Pulmonary TNF-α content was assayed using the Mouse TNF alpha ELISA Ready-SET-Go kit (eBiosciences) and the MPO content of the lungs was quantified by enzyme immunoassay (Cell Sciences). Lung homogenate samples were clarified by centrifugation, and assayed undiluted as per manufacturer's recommendations. Results of MPO, LDH and TNF-α determination were normalized for differences in fungal burden by multiplying values by the ratio of the GM content of lungs infected with the experimental strain divided by the GM content of lungs infected with wild-type Af293.

### Ethics statement

The mouse studies were carried out in accordance with the National Institutes of Health guidelines for the ethical treatment of animals. This protocol was approved by the Institutional Animal Care and Use Committee (IACUC) of the Los Angeles Biomedical Research Institute at Harbor-UCLA Medical Center (Animal Welfare Assurance Number A3330-01).

## Supporting Information

Figure S1 **The Δ** ***uge3*** **mutant exhibits normal growth under a variety of conditions.** Radial growth of hyphae of each of the indicated strains was determined under the indicated growth conditions. No significant difference between strains was observed in response to hypoxia, changes in pH, or low iron conditions.
(TIF)

Table S1 **PCR primers used in this study.**
(DOC)
