# Supplementary material for: Aspergillus Galactosaminogalactan Mediates Adherence to Host Constituents and Conceals Hyphal β-Glucan from the Immune System
Source: PLoS Pathog. 2013 Aug 22;9(8):e1003575. doi: 10.1371/journal.ppat.1003575 (PMC3749958; doi:10.1371/journal.ppat.1003575)
Supplement: Table S1 — PCR primers used in this study. (DOC) [file ppat.1003575.s002.doc]

| Primer name | Target gene | Sequence 5’-3’ |
| --- | --- | --- |
| U1 | *uge3* | CACCAAGGTTTACTATTTCTAGTGGATGG |
| U2 | *uge3* | CGAGGACGATATGCTCAGG |
| U3 | *uge3* | CACCTTTTCATTTTGGTATGGCGT |
| U4 | *uge3* | CGTGTCTGCTTTGGACTTGA |
| U5 | *uge3* | CGCTCGATATTACGACCTGAGAATG |
| U-ext1 | *uge3* | GCAGGCACCGTCGATTGCTC |
| U-ext4 | *uge3* | CCGTTGTTGACTTCCCGCGT |
| U-OE1 | *uge3* | GGGCCATGGATATCATGGACAGCTACCAGCAAT |
| U-OE2 | *uge3* | GGGTCCACTAGTCTAAGTAGATAACCCACTGAC |
| U-RT sense | *uge3* | GCTGTTAGCCTCCCAGTACC |
| U-RT antisense | *uge3* | GGACTTGGTCGTACCCCAT |
| HY | *hph* | CAACCACGGCCTCCAGAAGAAGA |
| YG | *hph* | GCGAGAGCCTGACCTATTGCATCT |
| tef1-RT sense | *tef1* | CCATGTGTGTCGAGTCCTTC |
| tef1-RT antisense | *tef1* | GAACGTACAGCAACAGTCTGG |
| 18S forward | 18S | GGCCCTTAAATAGCCCGGT |
| 18S reverse | 18S | TGAGCCGATAGTCCCCCTAA |
| 18S probe | 18S | 6-FAM-AGCCAGCGGCCCGCAAATG-MGB |
